# SVM-RFE Based Feature Selection and Taguchi Parameters Optimization for Multiclass SVM Classifier

**DOI:** 10.1155/2014/795624

**Published:** 2014-09-10

**Authors:** Mei-Ling Huang, Yung-Hsiang Hung, W. M. Lee, R. K. Li, Bo-Ru Jiang

**Affiliations:** ^1^Department of Industrial Engineering and Management, National Chin-Yi University of Technology, No. 57, Sec. 2, Zhong-Shan Road, Taiping District, Taichung 41170, Taiwan; ^2^Department of Industrial Engineering & Management, National Chiao-Tung University, No. 1001, Ta-Hsueh Road, Hsinchu 300, Taiwan

## Abstract

Recently, support vector machine (SVM) has excellent performance on classification and prediction and is widely used on disease diagnosis or medical assistance. However, SVM only functions well on two-group classification problems. This study combines feature selection and SVM recursive feature elimination (SVM-RFE) to investigate the classification accuracy of multiclass problems for Dermatology and Zoo databases. Dermatology dataset contains 33 feature variables, 1 class variable, and 366 testing instances; and the Zoo dataset contains 16 feature variables, 1 class variable, and 101 testing instances. The feature variables in the two datasets were sorted in descending order by explanatory power, and different feature sets were selected by SVM-RFE to explore classification accuracy. Meanwhile, Taguchi method was jointly combined with SVM classifier in order to optimize parameters *C* and *γ* to increase classification accuracy for multiclass classification. The experimental results show that the classification accuracy can be more than 95% after SVM-RFE feature selection and Taguchi parameter optimization for Dermatology and Zoo databases.

## 1. Introduction

The support vector machine (SVM) is one of the important tools of machine learning. The principle of SVM operation is as follows: a given group of classified data is trained by the algorithm to obtain a group of classification models, which can help predict the category of the new data [1, 2]. Its scope of application is widely used in various fields, such as disease or medical imaging diagnosis [3–5], financial crisis prediction [6], biomedical engineering, and bioinformatics classification [7, 8]. Although SVM is an efficient machine learning method, its classification accuracy requires further improvement in the case of multidimensional space classification and dataset for feature interaction variables [9]. Regarding such problems, in general, feature selection can be applied to reduce data structure complexity in order to identify important feature variables as a new set of testing instances [10]. By feature selection, inappropriate, redundant, and noise data of each problem can be filtered to reduce the computational time of classification and improve classification accuracy. The common methods of feature selection include backward feature selection (BFS), forward feature selection (FFS), and ranker [11]. Another feature selection method, support vector machine recursive feature elimination (SVM-RFE), can filter relevant features and remove relatively insignificant feature variables in order to achieve higher classification performance [12]. The research findings of Harikrishna et al. have shown that computation is simpler and can more effectively improve classification accuracy in the case of datasets after SVM-REF selection [13–15].

As SVM basically applies on two-class data [16], many scholars have explored the expansion of SVM on multiclass data [17–19]. However, classification accuracy is not ideal. There are many studies on choosing kernel parameters for SVM [20–22]. Therefore, this study applies SVM-RFE to sort the 33 variables for Dermatology dataset and 16 variables for Zoo dataset by explanatory power in descending order and selects different feature sets before using the Taguchi parameter design to optimize Multiclass SVM parameters  *C* and *γ* to improve the classification accuracy for SVM multiclass classifier.

This study is organized as follows.  Section 2 describes the research data; Section 3 introduces methods used through this paper; Section 4 discusses the experiment and results. Finally, Section 5 presents our conclusions.

## 2. Study Population

This study used the Dermatology dataset from University of California at Irvine (UCI) and the Zoo database from its College of Information Technology and Computers to conduct experimental tests, parameter optimization, and classification accuracy performance evaluation, using the SVM classifier.

In medicine, dermatological diseases are diseases of the skin that have a serious impact on health. As frequently occurring types of diseases, there are more than 1000 kinds of dermatological diseases, such as psoriasis, seborrheic dermatitis, lichen planus, pityriasis, chronic dermatitis, and pityriasis rubra pilaris. The Dermatology dataset was established by Nilsel in 1998 and contains 33 feature variables and 1 class variable (6-class).

The dermatology feature variables and data summary are as shown in Table 1. The Dermatology dataset has eight omissions. After removing the eight omissions, we retained 358 (instances) for this study. The instances of data of various categories are psoriasis (Class 1): 111 instances, seborrheic dermatitis (Class 2): 71 instances, lichen planus (Class 3): 60 instances, pityriasis (Class 4): 48 instances, chronic dermatitis (Class 5): 48 instances, and pityriasis rubra pilaris (Class 6): 20 instances. The Zoo dataset contains 17 Boolean-valued attributes and 101 instances. The instances of data of various categories are as follows: bear, and so forth (Class 1) 41 instances; chicken, and so forth (Class 2) 20 instances; seasnake, and so forth (Class 3) 5 instances; bass, and so forth (Class 4) 13 instances; (Class 5) 4 instances; frog, and so forth (Class 6) 8 instances; and honeybee, and so forth (Class 7) 10 instances.

Before feature selection, we conducted feature attribute coding. The feature attribute coding of Dermatology and Zoo databases is as shown in Tables 2 and 3.

## 3. Methodology

### 3.1. Research Framework

**Figure 1:**
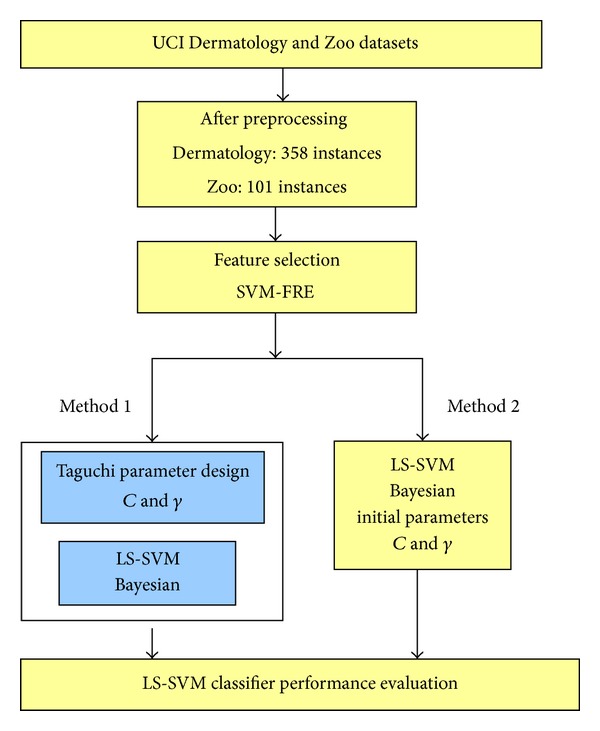
Research framework.

The research framework of the study is shown in Figure 1. Steps are as follows.

1. Database preprocessing: delete the omissions and feature variable coding for Dermatology and Zoo datasets. And there are 358 and 101 instances left for Dermatology and Zoo databases for further experiment, respectively.
2. Feature selection: apply SVM-RFE ranking according to the order of importance of the features, and determine the feature set that contributes to the classification.
3. Parameter optimization: apply Taguchi parameter design in the parameters (*C* & *γ*) optimization of a Multiclass SVM Classifier in order to enhance the classification accuracy for the multiclass dataset.

### 3.2. Feature Selection

Feature selection implies not only cardinality reduction, which means imposing an arbitrary or predefined cutoff on the number of attributes that can be considered when building a model, but also the choice of attributes, meaning that either the analyst or the modeling tool actively selects or discards attributes based on their usefulness for analysis. The feature selection method is a search strategy to select or remove some features of the original feature set to generate various types of subsets to obtain the optimum feature subset. The subsets selected each time are compared and analyzed according to the formulated assessment function. If the subset selected in step *m* + 1 is better than the subset selected in step *m*, the subset selected in step *m* + 1 can be selected as the optimum subset.

### 3.3. Linear Support Vector Machine (Linear SVM)

SVM is developed from statistical learning theory, as based on SRM (structural risk minimization). It can be applied on classification and nonlinear regression [6]. Generally speaking, SVM can be divided into linear SVM (linear SVM) and nonlinear SVM, described as follows.

(*1) Linear SVM.* The linear SVM encodes the training data of different types by classification with Class 1 as being “+1” and Class 2 as being “−1” and the mathematical symbol is {{*x*_*i*_, *y*_*i*_}_*i*−1_^*T*^, *x*_*i*_ ∈ *R* ^*m*^, *y*_*i*_ ∈ {−1, +1}}; the hyperplane is represented as follows:

(1) $$\begin{array}{c} w\cdot x+b=0, \end{array}$$

where *w* denotes weight vector, *x* denotes the input dataset, and *b* denotes a constant as a bias (displacement) in the hyperplane. The purpose of bias is to ensure that the hyperplane is in the correct position after horizontal movement. Therefore, bias is determined after training *w*. The parameters of the hyperplane include *w* and *b*. When SVM is applied on classification, the hyperplane is regarded as a decision function:

(2) $$\begin{array}{c} f\left(x\right)=\mathrm{sign}\left(w\cdot x+b\right). \end{array}$$

Generally speaking, the purpose of SVM is to obtain the hyperplane of the maximized marginal distance and improve the distinguishing function between the two categories of the dataset. The process of optimizing the distinguishing function of the hyperplane can be regarded as a quadratic programming problem:

(3) minimize  Lp=12||w||2subject  to yi(xi·w+b)−1≥0, i=1,…,l.

The original minimization problem is converted into a maximization problem by using the Lagrange Theory:

(4) max⁡    LD(α)=∑i=1lαi−12∑i=1l ∑j=1lαiαjyiyj(xixj)subject  to ∑i=1lαiyi=0, i=1,…,l        αi≥0, i=1,…,l.

Finally, the linear divisive decision making function is

(5) $$\begin{array}{c} f\left(x\right)=\mathrm{sign}\left(\overset{n}{\underset{i=1}{\sum }}y_{i}\alpha_{i}^{*}\left(x\cdot x_{i}\right)+b^{*}\right). \end{array}$$

If *f*(*x*) > 0, it means the sample is in the same category as samples marked with “+1”; otherwise, it is in the category of samples marked with “−1.” When the training data include noise, the linear hyperplane cannot accurately distinguish data points. By introducing slack variables *ξ*_*i*_ in the constraint, the original (3) can be modified into the following:

(6) minimize 12||w||2+C(∑i=1lξi)subject  to yi(xi·w+b)−1+ξi≥0, i=1,…,l       ξi≥0, i=1,…,l,

where *ξ*_*i*_ is the distance between the boundary and the classification point and penalty parameter *C* represents the cost of the classification error of training data during the learning process, as determined by the user. When *C* is greater, the margin will be smaller, indicating that the fault tolerance rate will be smaller when a fault occurs. Otherwise, when *C* is smaller, the fault tolerance rate will be greater. When *C* → *∞*, the linear inseparable problem will degenerate into a linear separable problem. In this case, the solution of the above mentioned optimization problem can be applied to obtain the various parameters and optimum solution of the target function using the Lagrangian coefficient; thus, the linear inseparable dual optimization problem is as follows:

(7) Max⁡     LD(α)=∑i=1lαi−12∑i=1l ∑j=1lαiαjyiyj(xixj)Subject  to ∑i=1lαiyi=0, i=1,…,l        0≤αi≤C, i=1,…,l.

Finally, the linear decision-making function is

(8) $$\begin{array}{c} f\left(x\right)=\mathrm{sign}\left(\overset{n}{\underset{i=1}{\sum }}y_{i}\alpha_{i}^{*}\left(x\cdot x_{i}\right)+b^{*}\right). \end{array}$$

(*2) Nonlinear Support Vector Machine (Nonlinear SVM).* When input training samples cannot be separated using linear SVM, we can use conversion function *φ* to convert the original 2-dimensional data into a new high-dimensional feature space for linear separable problem. SVM can efficiently perform a nonlinear classification using what is called the kernel trick, implicitly mapping their inputs into high-dimensional feature spaces. Presently, many different core functions have been proposed. Using different core functions regarding different data features can effectively improve the computational efficiency of SVM. The relatively common core functions include the following four types:

(1) linear kernel function:
  (9) $$\begin{array}{c} K\left(x_{i},y_{i}\right)=x_{i}^{t}\cdot y_{j}, \end{array}$$
(2) polynomial kernel function:
  (10) $$\begin{array}{c} K\left(x_{i},y_{j}\right)={\left(\gamma x_{i}^{t}x_{j}+r\right)}^{m},\;\gamma >0, \end{array}$$
(3) radial basis kernel function:
  (11) $$\begin{array}{c} K\left(x_{i},y_{j}\right)=\exp\left(\frac{-{||x_{i}-y_{j}||}^{2}}{2\sigma^{2}}\right),\;\gamma >0, \end{array}$$
(4) sigmoid kernel function:
  (12) $$\begin{array}{c} K\left(x_{i},y_{j}\right)=\tanh\left(\gamma x_{i}^{t}\cdot y_{j}+r\right), \end{array}$$

where the emissive core function is more frequently applied in high feature dimensional and nonlinear problems, and the parameters to be set are *γ* and *C*, which can slightly reduce SVM complexity and improve calculation efficiency; therefore, this study selects the emissive core function.

### 3.4. Support Vector Machine Recursive Feature Elimination (SVM-RFE)

A feature selection process can be used to remove terms in the training dataset that are statistically uncorrelated with the class labels, thus improving both efficiency and accuracy. Pal and Maiti (2010) provided a supervised dimensionality reduction method. The feature selection problem has been modeled as a mixed 0-1 integer program [23]. Multiclass Mahalanobis-Taguchi system (MMTS) is developed for simultaneous multiclass classification and feature selection. The important features are identified using the orthogonal arrays and the signal-to-noise ratio and are then used to construct a reduced model measurement scale [24]. SVM-RFE is an SVM-based feature selection algorithm created by [12]. Using SVM-RFE, Guyon et al. selected key and important feature sets. In addition to reducing classification computational time, it can improve the classification accuracy rate [12]. In recent years, many scholars improved the classification effect in medical diagnosis by taking advantage of this method [22, 25].

### 3.5. Multiclass SVM Classifier

SVM's basic classification principle is mainly based on dual categories. Presently, there are three main methods, one-against-all, one-against-one, and directed acyclic graph, to process multiclass problems [26], described as follows. 

(*1) One-Against-All (OAA).* Proposed by Bottou et al., (1994) the one-versus-rest converts the classification problem of *k* categories into *k* dual-category problems [27]. Scholars have also proposed subsequent effective classification methods [28]. In the training process, it must train *k* dual-category SVMs. When training the *i*th classifier, data in the *i*th category is regarded as “+1” and the data of the remaining categories is regarded as “−1” to complete the training of *k* dual-category SVM; during the testing process, each testing instance is tested by trained *k* dual-category SVMs. The classification results can be determined by comparing the outputs of SVM. Regarding unknown category *x*, the decision function arg max_*i*=1,…,*k*_(*w* ^*i*^)^*t*^ *ϕ*(*x*) + *b* ^*i*^ can be applied to generate *k* decision-making values, and category *x* is the category of the maximum decision making value. 

(*2) One-Against-One (OAO).* When there are *k* categories, two categories can produce an SVM; thus, it can produce *k*(*k* − 1)/2 classifiers and determine the category of the samples by a voting strategy [28]. For example, if there are three categories (1, 2, and 3) and a sample to be classified with an assumed category of 2, the sample will then be input into three SVMs. Each SVM will determine the category of the sample using decision making function sign⁡((*w* ^*ij*^)^*t*^Φ(*x*) + *b* ^*ij*^) and adds 1 to the votes of the category. Finally, the category with the most votes is the category of the sample. 

(*3) Directed Acyclic Graph (DAG).* Similar to OAO method, DAG is to disintegrate the classification problem *k* categories into a *k*(*k* − 1)/2 dual-category classification problem [18]. During the training process, it selects any two categories from *k* categories as a group, which it combines into a dual-category classification SVM; during the testing process, it establishes a dual-category acyclic graph. The data of an unknown category is tested from the root nodes. In a problem with *k* classes, a rooted binary DAG has *k* leaves labeled by the classes where each of the *k*(*k* − 1)/2 internal nodes is labeled with an element of a Boolean function [19].

## 4. Experiment and Results

### 4.1. Feature Selection Based on SVM-RFE

The main purpose of SVM-RFE is to compute the ranking weights for all features and sort the features according to weight vectors as the classification basis. SVM-RFE is an iteration process of the backward removal of features. Its steps for feature set selection are shown as follows.

1. Use the current dataset to train the classifier.
2. Compute the ranking weights for all features.
3. Delete the feature with the smallest weight.

Implement the iteration process until there is only one feature remaining in the dataset; the implementation result provides a list of features in the order of weight. The algorithm will remove the feature with smallest ranking weight, while retaining the feature variables of significant impact. Finally, the feature variables will be listed in the descending order of explanatory difference degree. SVM-RFE's selection of feature sets can be mainly divided into three steps, namely, (1) the input of the datasets to be classified, (2) calculation of weight of each feature, and (3) the deletion of the feature of minimum weight to obtain the ranking of features. The computational step is shown as follows [12].

(*1) Input*

 Training sample: *X*_0_ = [*x*_1_,*x*_2_,…,*x*_*m*_]^*T*^.
 Category: *y* = [*y*_1_,*y*_2_,…,*y*_*m*_]^*T*^.
 The current feature set: *s* = [1,2,…, *n*].
 Feature sorted list: *r* = [].

(*2) Feature Sorting*

 Repeat the following process until *s* = [].
 To obtain the new training sample matrix according to the remaining features: *X* = *X*_0_(:, *s*).
 Training classifier: *α* = SVM-train(*X*, *y*).
 Calculation of weight: *w* = ∑_*k*_ *α*_*k*_ *y*_*k*_ *x*_*k*_.
 Calculation of sorting standards: *c*_*i*_ = (*w*_*i*_)^2^.
 Finding the features of the minimum weight: *f* = arg min⁡(*c*).
 Updating feature sorted list: *r* = [*s*(*f*), *r*].
 Removing the features with minimum weight: *s* = *s*(1 : −1, *f* + 1 : length(*s*)).

(*3) Output: Feature Sorted List r*. In each loop, the feature with minimum (*w*_*i*_)^2^ will be removed. The SVM then retrains the remaining features to obtain the new feature sorting. SVM-RFE repeatedly implements the process until obtaining a feature sorted list. Through training SVM using the feature subsets of the sorted list and evaluating the subsets using the SVM prediction accuracy, we can obtain the optimum feature subsets.

### 4.2. SVM Parameters Optimization Based on Taguchi Method

Taguchi Method rises from the engineering technological perspective and its major tools include the orthogonal array and *SN* ratio, where *SN* ratio and loss function are closely related. A higher *SN* ratio indicates fewer losses [29]. Parameter selection is an important step of the construction of the classification model using SVM. The differences in parameter settings can affect classification model stability and accuracy. Hsu and Yu (2012) combined Taguchi method and Staelin method to optimize the SVM-based e-mail spam filtering model and promote spam filtering accuracy [30]. Taguchi parameter design has many advantages. For one, the effect of robustness on quality is great. Robustness reduces variation in parts by reducing the effects of uncontrollable variation. More consistent parts are equal to better quality. Also, the Taguchi method allows for the analysis of many different parameters without a prohibitively high amount of experimentation. It provides the design engineer with a systematic and efficient method for determining near optimum design parameters for performance and cost. Therefore, by using the Taguchi quality parameter design, this study conducts the optimization design of parameters *C* and *γ* to enhance the accuracy of SVM classifier on the diagnosis of multiclass diseases.

This study uses the multiclass classification accuracy as the quality attribute of the Taguchi parameter design [21]. In general, when the classification accuracy is higher, it means the accuracy of the classification model is better; that is, the quality attribute is larger-the-better (LTB), and *SN*_LTB_ is defined as:

(13) $$\begin{array}{c} SN_{\text{LTB}}=-10\;\text{lo}{\text{g}}_{10}\left(MSD\right)=-10\;\text{lo}{\text{g}}_{10}\left[\frac{1}{n}\overset{n}{\underset{i=1}{\sum }}\frac{1}{y_{i}^{2}}\right]. \end{array}$$

### 4.3. Evaluation of Classification Accuracy

Cross-validation measurement divides all the samples into a training set and a testing set. The training set is the learning data of the algorithm to establish the classification rules; the samples of the testing data are used as the testing data to measure the performance of the classification rules. All the samples are randomly divided into *k*-folds by category, and the data are mutually repelled. Each fold of the data is used as the testing data and the remaining *k* − 1 folds are used as the training set. The step is repeated *k* times, and each testing set validates the classification rules learnt from the corresponding training set to obtain an accuracy rate. The average of the accuracy rates of all *k* testing sets can be used as the final evaluation results. The method is known as *k*-fold cross-validation.

### 4.4. Results and Discussion

The ranking order of all features for Dermatology and Zoo* databases*, using RFE-SVM, is summarized as follows: Dermatology = {V1, V16, V32, V28, V19, V3, V17, V2, V15, V21, V26, V13, V14, V5, V18, V4, V23, V11, V8, V12, V27, V24, V6, V25, V30, V29, V10, V31, V22, V20, V33, V7, V9} and Zoo = {V13, V9, V14, V10, V16, V4, V8, V1, V11, V2, V12, V5, V6, V3, V15, V7}. According to the suggestions of scholars, the classification error rate of OAO is relatively lower when the number of testing instances is below 1000. Multiclass SVM parameter settings can affect the Multiclass SVM's classification accuracy. Arenas-García and Pérez-Cruz applied SVMs' parameters setting in the multiclass Zoo dataset [31]. They have carried out simulation, using Gaussian kernels, for all possible combinations of *C* and* Garmar* from *C* = [*l*, 3,10,30,100] and* Garmar* = sqrt(0.25d), sqrt(0.5d), sqrt(d), sqrt(2d), and sqrt(4d) with d being the dimension of the input data. In this study, we have executed wide ranges of the parameter settings for Dermatology and Zoo databases. Finally, the parameter settings are suggested as Dermatology (*C*, *γ*) = {*C* = 1,10,50,100  and  *γ* = 1,3, 10,12}, Zoo (*C*, *γ*) = {*C* = 1,10,50,100  and  *γ* = 0.1,5, 10,12}, and the testing accuracies are shown in Table 4.

As shown in Table 4, regarding parameter *C*, when *C* = 10 and *γ* = {5,10,12}, the accuracy of the experiment is higher than that of the experimental combination of *C* = 1 and *γ* = {5,10,12}; moreover, regarding parameter *γ*, the experimental accuracy rate in the case of *γ* = 5 and *C* = {1,10,50,100} is higher than that of the experimental combination of *γ* = 0.1 and *C* = {1,10,50,100}. The near optimal value of *C* or *γ* may not be the same for different databases. Finding the appropriate parameter settings is important for the performance of classifiers. Practically, it is impossible to simulate every possible combination of parameter settings. And that is the reason why Taguchi methodology is applied to reduce the experimental combinations for SVM. The experimental step used in this study was first referred to the related study, ex, *C* = [1,3, 10,30,100], [31]; then set a possible range for both databases (*C* = 1~100, *γ* = 1~12). After that, we slightly adjusted the ranges to understand if there will be better results in Taguchi quality engineering parameter optimization for each database. According to our experimental result, the final parameter settings *C* and *γ* range 10~100 and 2.4~10, respectively, for Dermatology database; the parameters settings *C* and *γ* range 5~50 and 0.08~11, respectively, for Zoo databases. Within the range of Dermatology and Zoo databases parameters *C* and *γ*, we select three parameter levels and two control factors, *A* and *B*, to represent parameters *C* and *γ*, respectively. The Taguchi orthogonal array experiment selects *L*_9_(3^2^) and the factor level configuration is as illustrated in Table 5.

After data preprocessing, Dermatology and Zoo databases include 358 and 101 testing instances, respectively. The various experiments of the orthogonal array are repeated five times (*n* = 5); the experimental combination and observations are summarized, as shown in Tables 6 and 7. According to (13), we can calculate the *SN* ratio for Taguchi experimental combination #1 as

(14) SNLTB=−10 log10[15×(10.96312+10.97012+10.96972            +10.96272+10.96142)]=−0.3060.

The calculation results of the *SN* ratios of the remaining eight experimental combinations are summarized, as in Table 6. The Zoo experimental results and *SN* ratio calculation are as shown in Table 7. According to the above results, we then calculate the average *SN* ratios of the various factor levels. With the experiment of Table 8 as an example, the average *SN* ratio ${\overset{-}{A}}_{1}$ of Factor *A* at Level 1 is

(15) $$\begin{array}{c} {\bar{A}}_{1}=\frac{1}{3}\left[-0.3060+\left(-0.2755\right)+\left(-0.1647\right)\right]=-0.2487. \end{array}$$

**Figure 2:**
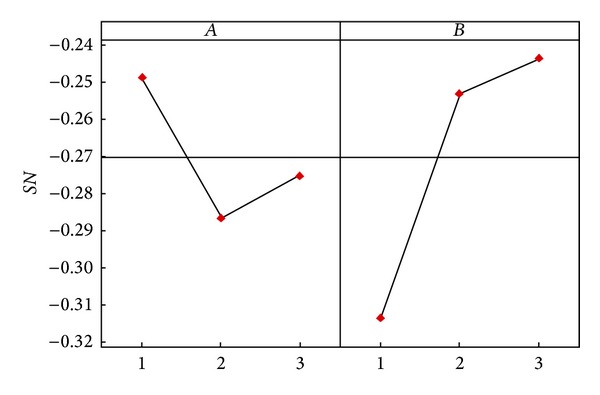
Main effect plots for *SN* ratio of Dermatology database.

**Figure 3:**
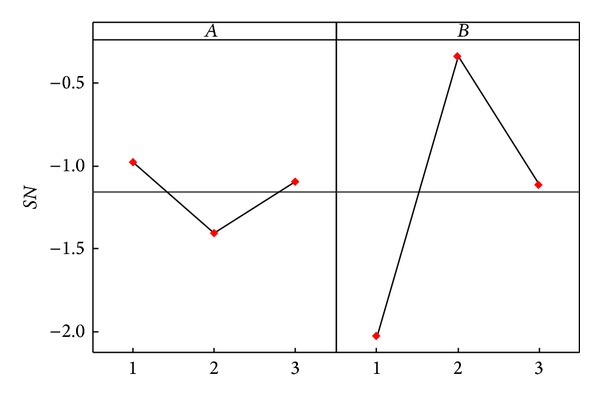
Main effect plots for *SN* ratio of Zoo database.

Similarly, we can calculate the average effects of ${\overset{-}{A}}_{2}$ and ${\overset{-}{A}}_{3}$ from Table 6. The difference analysis results of the various factor levels of Dermatology and Zoo databases are as shown in Table 8. The factor effect diagrams are as shown in Figures 2 and 3. As a greater *SN* ratio represents better quality, according to the factor level difference and factor effect diagrams, the Dermatology parameter level combination is *A*_1_ *B*_3_; in other words, parameters *C* = 10, *γ* = 10,* Zoo* parameter level combination is *A*_1_ *B*_2_, and the parameter settings are *C* = 5, *γ* = 4.

**Figure 4:**
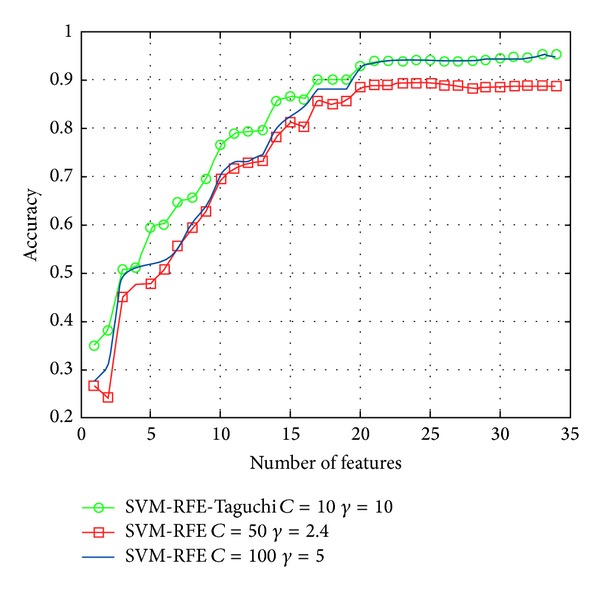
Classification performance comparison of Dermatology database.

When constructing the Multiclass SVM model using SVM-RFE, three different feature sets are selected according to their significance. At the first stage, Taguchi quality engineering is applied to select the optimum values of parameters *C* and *γ*. At the second stage, it constructs the Multiclass SVM Classifier and compares the classification performance according to the above parameters. In the Dermatology experiment, Table 9 illustrates the two feature subsets containing 23 and 33 feature variables. The 33 feature sets are tested by SVM and SVM, as based on Taguchi. The parameter settings and testing accuracy rate results are as shown in Table 9. The experimental results, as shown in Figure 4, show that the SVM (*C* = 10, *γ* = 10) testing accuracy rate of the 17-feature sets datasets can be higher than 90%, which is better than the accuracy rate of 20-feature sets dataset SVM (*C* = 10, *γ* = 11), up to 90%. Moreover, regardless of how many sets of feature variables are selected, the accuracy of SVM (*C* = 50, *γ* = 2.4) cannot be higher than 90%.

**Figure 5:**
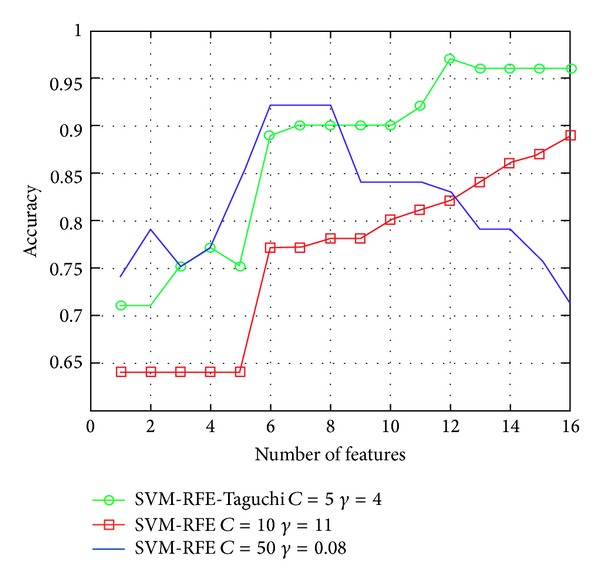
Classification performance comparison of Zoo database.

Regarding the Zoo experiment, Table 10 summarizes the experimental test results of sets containing 6, 12, and 16 feature variables using SVM and SVM based on Taguchi. As shown in Table 10, the experimental results show that the classification accuracy rate of the set of 12-feature variables in the classification experiment using SVM-RFE-Taguchi (*C* = 10, *γ* = 10) is the highest, up to 97% ± 0.0396. As shown in Figure 5, the experimental results show that the classification accuracy rate of the dataset containing 7 feature variables by SVM-RFE-Taguchi (*C* = 50, *γ* = 2.4) can be higher than 90%, which can obtain relatively better prediction effects.

## 5. Conclusions

As the study on the impact of feature selection on the multiclass classification accuracy rate becomes increasingly attractive and significant, this study applies SVM-RFE and SVM in the construction of a multiclass classification method in order to establish the classification model. As RFE is a feature selection method of a wrapper model, it requires a previously defined classifier as the assessment rule of feature selection; therefore, SVM is used as the RFE assessment standard to help RFE in the selection of feature sets.

According to the experimental results of this study, with respect to parameter settings, the impact of parameter selection on the construction of SVM classification model is huge. Therefore, this study applies the Taguchi parameter design in determining the parameter range and selection of the optimum parameter combination for SVM classifier, as it is a key factor influencing the classification accuracy. This study also collected the experimental results of using different research methods in the case of Dermatology and Zoo databases [16, 32, 33], as shown in Table 11. By comparison, the proposed method can achieve higher classification accuracy.

## Figures and Tables

**Table 1:** Feature information for Dermatology and Zoo databases.

	Dermatology	Zoo
Dataset characteristics	Multivariate	Multivariate
Attribute characteristics	Categorical, integer	Categorical, integer
Associated tasks	Classification	Classification
Area	Life	Life
Number of instances	366	101
Number of attributes	33	16
Number of class	6	7

**Table 2:** Attributes of Dermatology database.

ID	Attribute
V1	Erythema
V2	Scaling
V3	Definite borders
V4	Itching
V5	Koebner phenomenon
V6	Polygonal papules
V7	Follicular papules
V8	Oral mucosal involvement
V9	Knee and elbow involvement
V10	Scalp involvement
V11	Family history
V12	Melanin incontinence
V13	Eosinophils in the infiltrate
V14	PNL infiltrate
V15	Fibrosis of the papillary dermis
V16	Exocytosis
V17	Acanthosis
V18	Hyperkeratosis
V19	Parakeratosis
V20	Clubbing of the rete ridges
V21	Elongation of the rete ridges
V22	Thinning of the suprapapillary epidermis
V23	Spongiform pustule
V24	Munro microabscess
V25	Focal hypergranulosis
V26	Disappearance of the granular layer
V27	Vacuolisation and damage of basal layer
V28	Spongiosis
V29	Saw-tooth appearance of retes
V30	Follicular horn plug
V31	Perifollicular parakeratosis
V32	Inflammatory mononuclear infiltrate
V33	Band-like infiltrate
V34	Age

**Table 3:** Attributes of Zoo database.

ID	Attribute
V1	Hair
V2	Feathers
V3	Eggs
V4	Milk
V5	Airborne
V6	Aquatic
V7	Predator
V8	Toothed
V9	Backbone
V10	Breathes
V11	Venomous
V12	Fins
V13	Legs
V14	Tail
V15	Domestic
V16	Cat-size

**Table 4:** Classification accuracy comparison.

Dermatology database					Zoo database				
*C*	*γ*				*C*	*γ*			
	1	3	10	12		0.1	5	10	12
1	52.57%	95.18%	94.08%	94.22%	1	71.18%	78.09%	62.36%	40.64%
10	52.57%	96.04%	97.94%	97.93%	10	71.18%	96.00%	91.00%	85.09%
50	52.57%	96.31%	96.86%	96.58%	50	71.18%	96.09%	96.00%	96.00%
100	52.57%	96.31%	96.32%	96.03%	100	71.18%	96.09%	96.09%	96.00%

**Table 5:** Factor level configuration of LS-SVM parameter design.

Dermatology database				Zoo database			
Control factor	Level			Control factor	Level		
	1	2	3		1	2	3
*A*(*C*)	10	50	100	*A*(*C*)	5	10	50
*B*(*γ*)	2.4	5	10	*B*(*γ*)	0.08	4	11

**Table 6:** Summary of experiment data of Dermatology database.

Number	Control factor		Observation					Average	*SN *
	*A*	*B*	*y*_1_	*y*_2_	*y*_3_	*y*_4_	*y*_5_		
1	1	1	0.9631	0.9701	0.9697	0.9627	0.9614	0.9654	−0.3060
2	1	2	0.9686	0.9749	0.9653	0.9621	0.9732	0.9688	−0.2755
3	1	3	0.9795	0.9847	0.9848	0.9838	0.9735	0.9813	−0.1647
4	2	1	0.9630	0.9615	0.9581	0.9599	0.9668	0.9619	−0.3379
5	2	2	0.9687	0.9721	0.9704	0.9707	0.9626	0.9689	−0.2746
6	2	3	0.9685	0.9748	0.9744	0.9712	0.9707	0.9719	−0.2475
7	3	1	0.9671	0.9689	0.9648	0.9668	0.9645	0.9664	−0.2967
8	3	2	0.9741	0.9704	0.9797	0.9799	0.9767	0.9762	−0.2098
9	3	3	0.9625	0.9633	0.9642	0.9678	0.9619	0.9639	−0.3191

(*A*_1_ = 10, *A*_2_ = 50, *A*_3_ = 100; *B*_1_ = 2.4, *B*_2_ = 5, *B*_3_ = 10).

**Table 7:** Summary of experiment data of Zoo database.

Number	Control factor		Observation					Average	*SN *
	*A*	*B*	*y*_1_	*y*_2_	*y*_3_	*y*_4_	*y*_5_		
1	1	1	0.9513	0.9673	0.9435	0.9567	0.9546	0.9547	−0.4037
2	1	2	0.9600	0.9616	0.9588	0.9611	0.9608	0.9605	−0.3504
3	1	3	0.7809	0.7833	0.7820	0.7679	0.7811	0.7790	−2.1694
4	2	1	0.7118	0.6766	0.7368	0.7256	0.7109	0.7123	−2.9571
5	2	2	0.9600	0.9612	0.9604	0.9519	0.9440	0.9555	−0.3960
6	2	3	0.8900	0.8947	0.9214	0.9050	0.9190	0.9060	−0.8598
7	3	1	0.7118	0.7398	0.7421	0.7495	0.7203	0.7327	−2.7064
8	3	2	0.9610	0.9735	0.9709	0.9752	0.9661	0.9693	−0.2709
9	3	3	0.9600	0.9723	0.9707	0.9509	0.9763	0.9660	−0.3013

(*A*_1_ = 5, *A*_2_ = 10, *A*_3_ = 50; *B*_1_ = 0.08, *B*_2_ = 4, *B*_3_ = 11).

**Table 8:** Average of each factor at all levels.

Dermatology					Zoo				
Control factor	Level				Control factor	Level			
	1	2	3	Difference		1	2	3	Difference
*A*(*C*)	−0.2487	−0.2867	−0.2752	0.0380	*A*(*C*)	−0.9745	−1.4043	−1.0929	0.4298
*B*(*γ*)	−0.3135	−0.2533	−0.2438	0.0697	*B*(*γ*)	−2.0224	−0.3391	−1.1102	1.6833

**Table 9:** Classification performance comparison of Dermatology database.

Methods	Dimensions	*C*	*γ*	Accuracy
SVM	33	100	5	95.10% ± 0.0096
SVM-RFE	23	50	2.4	89.28% ± 0.0139
SVM-RFE-Taguchi	23	10	10	95.38% ± 0.0098

**Table 10:** Classification performance comparison of Zoo database.

Methods	Dimensions	*C*	*γ*	Accuracy
SVM	16	10	11	89% ± 0.0314
SVM-RFE	6	50	0.08	92% ± 0.0199
SVM-RFE-Taguchi	12	5	4	97% ± 0.0396

**Table 11:** Comparison of classification accuracy in related literature.

Author	Method	Accuracy%
Dermatology database		
Xie et al. (2005) [16]	FOut_SVM	91.74%
Srinivasa et al. (2006) [32]	FCM_SVM	83.30%
Ren et al. (2006) [33]	LDA_SVM	72.09%
Our Method (2014)	SVM-RFE-Taguchi	95.38%

Zoo database		
Xie et al. (2005) [16]	FOut_SVM	88.24%
He (2006) [34]	NFPH_k-modes	92.08%
Golzari et al. (2009) [35]	Fuzzy_AIRS	94.96%
Our Method (2014)	SVM-RFE-Taguchi	97.00%
